# Nanoforging Single Layer MoSe_2_ Through Defect Engineering with Focused Helium Ion Beams

**DOI:** 10.1038/srep30481

**Published:** 2016-08-02

**Authors:** Vighter Iberi, Liangbo Liang, Anton V. Ievlev, Michael G. Stanford, Ming-Wei Lin, Xufan Li, Masoud Mahjouri-Samani, Stephen Jesse, Bobby G. Sumpter, Sergei V. Kalinin, David C. Joy, Kai Xiao, Alex Belianinov, Olga S. Ovchinnikova

**Affiliations:** 1 Center for Nanophase Materials Sciences, Oak Ridge National Laboratory, Oak Ridge, TN 37831, USA; 2 Department of Materials Science and Engineering, University of Tennessee, Knoxville, TN 37996, USA; 3 The Procter and Gamble Company Winton Hill Business Center (WBHC), Cincinnati, OH 45224, USA; 4 The Institute for Functional Imaging of Materials, Oak Ridge National Laboratory, Oak Ridge, TN 37931, USA; 5 Computer Science & Mathematics Division, Oak Ridge National Laboratory, Oak Ridge, TN 37831, USA

## Abstract

Development of devices and structures based on the layered 2D materials critically hinges on the capability to induce, control, and tailor the electronic, transport, and optoelectronic properties via defect engineering, much like doping strategies have enabled semiconductor electronics and forging enabled introduction the of iron age. Here, we demonstrate the use of a scanning helium ion microscope (HIM) for tailoring the functionality of single layer MoSe_2_ locally, and decipher associated mechanisms at the atomic level. We demonstrate He^+^ beam bombardment that locally creates vacancies, shifts the Fermi energy landscape and increases the Young’s modulus of elasticity. Furthermore, we observe for the first time, an increase in the *B*-exciton photoluminescence signal from the *nanoforged* regions at the room temperature. The approach for precise defect engineering demonstrated here opens opportunities for creating functional 2D optoelectronic devices with a wide range of customizable properties that include operating in the visible region.

Layered materials are broadly perceived as next generation components for scaling various electronic and information technology devices^1^,^2^,^3^. Two-dimensional transition-metal dichalcogenides (TMDCs) belong to the MX_2_ family where M = W, Mo, or Nb and X = Se, S, or Te^1^,^4^,^5^. An analogue of graphene, TMDCs are a class of layered compounds that exhibit a band gap, and strong in-plane bonding^4^; capable of being prepared by mechanical and liquid exfoliation^1^,^6^. Exfoliated TMDCs have already been implemented in thin-film transistors, electrostatically-gated light emitting diodes, and electrostatically-gated photodiodes^7^,^8^,^9^,^10^. Recently, CVD-grown, high quality 2D materials have exhibited viability in large-scale fabrication^11^,^12^. More importantly, TMDCs have a complementary range of properties to graphene, making mass produced heterogeneous 2D devices a distinct possibility^13^. Therefore, the focus is now shifting towards structural and functional tuning of 2D materials, akin to the current semiconductor efforts^11^,^12^. Molybdenum diselenide (MoSe_2_) is an indirect bandgap semiconductor (1.1 eV) in the bulk, transitioning to a direct bandgap semiconductor (1.5 eV) at a single layer^4^. While some band gap control has been demonstrated through the introduction of dopants and defects, these studies are generally performed on bulk samples with little local control at the level of an individual 2D flake^3^,^14^,^15^,^16^,^17^,^18^. The recent development of the helium ion microscope (HIM)^19^,^20^,^21^ as a single nanometer level precision tool for direct-write lithography^22^,^23^ and ion beam-induced deposition^24^,^25^, pushes the limits of nanofabrication by allowing finer local control over material structure without reactive ion implantation. Our earlier work demonstrated the ability to tune the graphene nanoribbon conductivity in a HIM^26^. Additionally, other work indicated the possibility of lateral tuning of material properties through the introduction of localized defects by HIM in graphene, WSe2, and MoS_2_^27^,^28^,^29^. However, a direct link in TMDCs between localized electronic changes and their manifestation in physical material behavior is yet to be established.

Herein, we discuss the use of a focused helium ion beam in tailoring the functionality of MoSe_2_ electronic devices with nanometer precision. Using a helium ion beam at high dose allows milling and structuring of MoSe_2_, while lower doses introduce defects thereby tuning mechanical, electromechanical and optoelectronic properties of the MoSe_2_. By coupling *nanoforging* in a HIM with scanning transmission electron microscopy (STEM), scanning probe microscopy (SPM), optical spectroscopy, and first-principles density functional theory (DFT) we are able to observe charge trapping at defect sites, evolution of material elasticity, and changes in Fermi levels. Additionally, we demonstrate, for the first time, the ability of the He^+^ beam to enhance the photoluminescence (PL) intensity of the *B*-exciton at room temperature leading to the potential of tuning the optical response of the material into the visible range.

## Results

**Figure 1:**
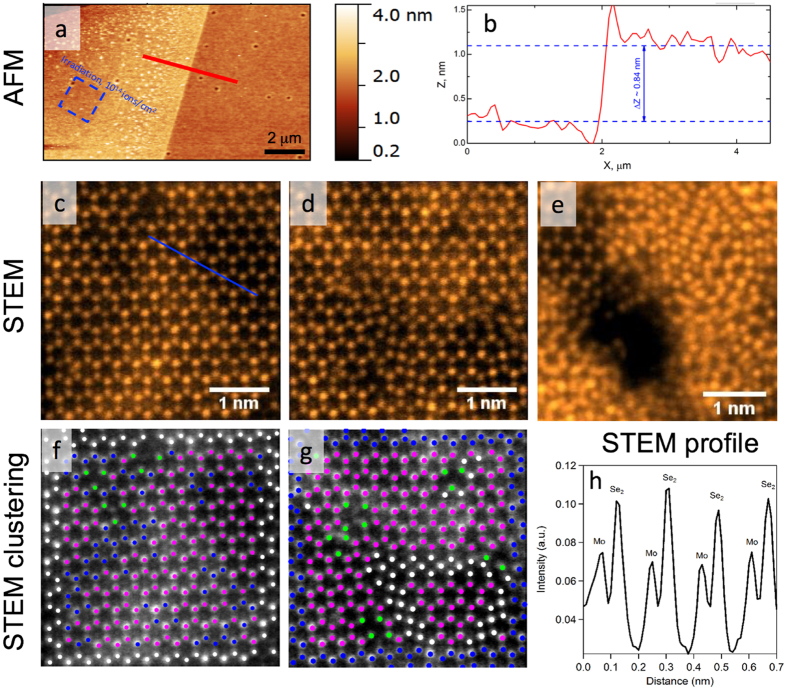
(**a**) AFM topography image of single layer supported MoSe_2_ flake and (**b**) its height profile as labeled on (**a**). (**c**–**e**) Atomic resolution MAADF-STEM images of (**c**) pristine region, (**d**) irradiated region at 1 × 10^15^ He^+^/cm^2^, and (**e**) 1 × 10^16^ He^+^/cm^2^. (**f**,**g**) *k*-means clustering STEM images in (**c**,**d**) color corresponds to the number of cluster. (**h**) Profile of pristine MoSe_2_ (**c**).

Functional tuning of mechanical, electromechanical and optoelectronic properties through defect engineering in single layer MoSe_2_ was investigated by irradiating suspended and supported samples with a focused He^+^ beam in a HIM. Thickness of the flake was measured using tapping-mode AFM (Fig. 1a,b). The range of the He^+^ dose was 1.0 × 10^14^ ions/cm^2^ to 1.0 × 10^16^ ions/cm^2^; with exposures above the upper limit resulting in complete amorphization of the material. To visualize the distribution and concentration of structural defects in suspended samples we utilized aberration-corrected STEM. The middle-angle annular dark field (MAADF) STEM images of single layer MoSe_2_ in Fig. 1(c–e) correspond to pristine, 1.0 × 10^15^ ions/cm^2^ and 1.0 × 10^16^ ions/cm^2^ dosed samples respectively. For identification of the Mo and Se atoms we used value of the STEM signal. A profile of the pristine sample (Fig. 1h) shows significant difference in the value of the signal for Mo and Se. Irradiation by 1.0 × 10^15^ ions/cm^2^ dose lead to formation of the numerous structural defects including Mo and Se vacancies (Fig. 1d), which can be attributed to knock-on damage caused by the energetic ion beam. Further increase in the dose by an order of magnitude (Fig. 1e), results in reduced crystallinity and a significant increase in the concentration of atomic defects.

A local crystallography approach was used in establishing regions with high defect concentration^30^,^31^,^32^. This approach performs k-means clustering on the detected atoms characterized by the set of parameters, including local bond lengths and angles of the neighboring atoms (see details in *Methods* section). This clustering allows splitting atoms on a few groups. For instance, clustering on the pristine sample (Fig. 1f) selects boundary atoms (labeled by white color), bulk atoms (blue and pink) and atoms neighboring to defects (green). Similar analysis performed on the data from the irradiated region (Fig. 1g) revealed a 17% increase in defect concentration. We were to able to apply the same approach to the 1.0 × 10^16^ ions/cm^2^ image due to the lack of significant crystallinity in captured images. In addition to quantify the number of atoms in pristine areas, our analysis allows us to explore the *types* of defects induced by the He^+^ beam. Specifically, defects can be classified by the number of missing atoms such as Se and Mo vacancies, differences in bond lengths in the damaged lattice and angles where neighbors are positioned, *etc*.

**Figure 2:**
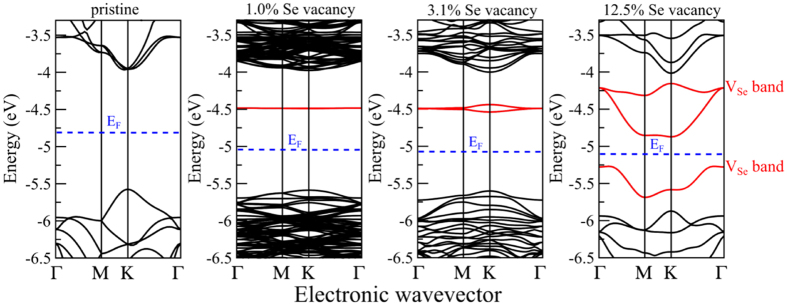
Calculated electronic band structures of single layer MoSe_2_ with different Se vacancy concentrations. All band energies are aligned to the vacuum potential for direct comparison. The vacancy induced in-gap bands are highlighted in red color. The Fermi level is set at the middle of the band gap for each system, as shown by the blue dash line.

To understand the role of defects on electronic properties of a single layer MoSe_2_, we first calculated DFT band structures of different vacancy concentrations (see Methods section for details). Although both Mo and Se vacancies can be observed by STEM, the majority of the vacancies correspond to Se vacancies. As reported previously^16^,^33^, the metal atoms require much higher energies to be knocked out since they are bonded to six chalcogenide neighbors, while the chalcogenide atoms, bonded to three metal neighbors, are relatively easier to be removed from the lattice and hence, our calculations were focused primarily on Se vacancies. Results of the modeling with Mo defects can be also found in Supplementary Materials (Fig. S1). Pristine, single layer MoSe_2_ has a direct band gap of ~1.6 eV at the *K* point and the introduction of a Se vacancy induces several defect bands in the band gap (denoted as V_Se_ bands and highlighted in red in Fig. 2). At relatively low vacancy concentrations (1.0% and 3.1%), there are two nearly degenerate in-gap V_Se_ bands above the Fermi level, which are almost dispersion-less; as their charge densities are well localized around vacancy sites, consistent with previous work on monolayer MoS_2_ with S vacancies^34^,^35^. As the vacancy concentration increases to 12.5%, the V_Se_ bands become more dispersive, since vacancy sites get closer and interact more strongly. An additional in-gap V_Se_ band also appears below the Fermi level (Fig. 2). Due to these in-gap V_Se_ bands, the Fermi levels of defect-rich systems are modified with respect to the pristine one.

**Figure 3:**
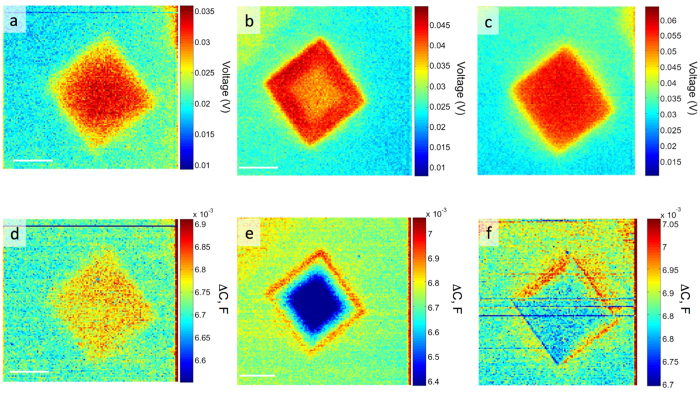
Tapping mode band excitation (BE) Kelvin probe force microscopy (KPFM) of supported MoSe_2_. (**a–c**) Local contact potential difference (LCPD) and (**d–f**) capacitance gradient maps of regions irradiated by He^+^ beam with different doses: (**a**,**d**) 1 × 10^14^ ions/cm^2^ (**b**,**e**) 1 × 10^15^ ions/cm^2^ and (**c**,**f**) 1 × 10^16^ ions/cm^2^.

The changes in the Fermi levels of the *nanoforged* MoSe_2_ were explored using Band-Excitation Kelvin Probe Microscopy (BE-KPFM)^36^,^37^,^38^. The difference in Fermi levels stems from trapped charges and uncompensated dangling bonds present after irradiation, as highlighted by the DFT calculations in Fig. 2. Increasing the dose of the ion beam in the MoSe_2_ leads to an increase in the concentration of structural defects, and by extension enrichment of trapped charges in the dosed regions, as was previously shown in the STEM images in Fig. 1c–e. Using BE-KPFM we were able to record the change in the surface potential, which is directly linked local work function and can be further linked to charge trapping at point defects^2^,^10^. Figure 3(a–c) shows the local contact potential difference (LCPD) between the cantilever and the sample; corresponding to a difference in Fermi levels in the irradiated regions by 1.0 × 10^14^ ions/cm^2^, 1.0 × 10^15^ ions/cm^2^ and 1.0 × 10^16^ ions/cm^2^ doses, respectively. Averaged changes in the LCPD show a small increase in the local work function with irradiation dose: 9 meV increase for 10^14^ ions/cm^2^ dose, 16 meV for 10^15^ ions/cm^2^ and 28 meV for 10^16^ ions/cm^2^. Furthermore, as the ion beam modifies the work function of the irradiated MoSe_2_, we observed changes in the capacitance gradient measured on the MoSe_2_ surface (Fig. 3d–f), which are related with He-ion induced change in the dielectric properties of the modified region. The measured trend of change in the local work function is in agreement with our DFT calculations, which show continuous downshift of the Fermi level with the increasing vacancy concentration (264 meV downshift at 1.0% vacancy, 293 meV downshift at 3.1% vacancy, and 300 meV downshift at 12.5% vacancy), due to the vacancy induced in-gap bands (see blue dashed lines in Fig. 2). Consequently, the work function of the defect-rich system is higher than that of the pristine one, and furthermore the work function slightly increases with the growing vacancy concentration, similar to the experimental observation that the work function increases slightly with the increasing dose.

**Figure 4:**
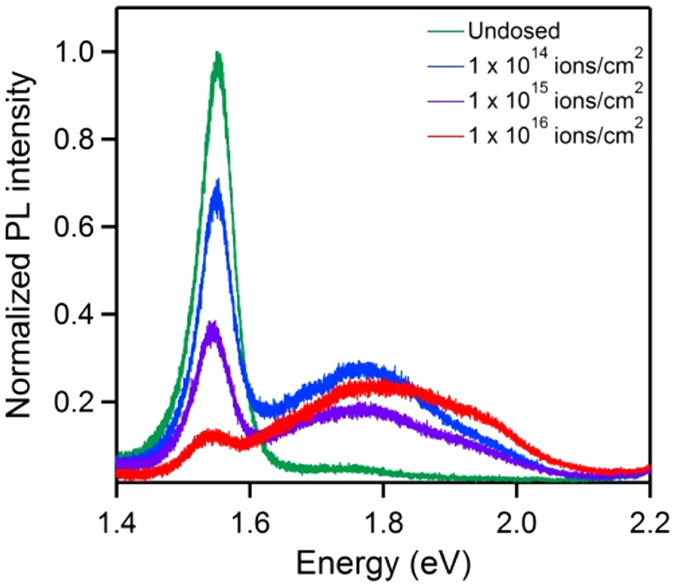
Photoluminescence (PL) spectra of supported MoSe_2_ indicating the evolution of the *A*-exciton (~1.55 eV) and *B*-exciton (~1.77 eV) peaks in undosed region (green trace) and He^+^ beam-irradiated regions corresponding to doses of 1 × 10^14^ ions/cm^2^ (blue trace), 1 × 10^15^ ions/cm^2^ (purple trace), and 1 × 10^16^ ions/cm^2^ (red trace).

To solidify findings related to the electronic structure modification by the HIM, we utilize photoluminescence (PL) spectroscopy measurements at room temperature^39^. Figure 4 is a normalized PL spectra from differently dosed regions within the single layer MoSe_2_ crystal. The green trace corresponds to a pristine region of the crystal and shows the characteristic peaks at ~1.55 eV and ~1.77 eV for the *A*- and *B*- excitons, respectively^40^. These excitons are due to vertical transitions at the *K* point of the Brillouin zone between the spin-orbit split valence band maximum (VBM-*A* and VBM-*B*) and the conduction band minimum (CBM)^40^,^41^,^42^. As the crystal is exposed to an increasing ion beam dose (blue, purple and red traces), the PL intensity of the *A*-exciton steadily decreases while interestingly the intensity of the *B-exciton* increases. Notably, significant difference in the intensity of B-*exciton* can be seen between pristine and irradiated regions (of any dose) only. Dependence of the B-*exciton* intensity on the dose is not stable, which can be attributed to charging during He^+^ implantation leading to minor fluctuations in substrate/film interaction. However, compared to the pristine region, the signal of the *B*-exciton is significantly enhanced in the He^+^ irradiated areas. Numerous studies investigated the effect of defects on the PL spectra of monolayer TMDCs^2^,^43^,^44^, but have focused on the *A*-exciton peak or new defect-induced peaks at lower energies relative to the *A*-exciton peak. In our experiments, we expect that the decrease in the intensity of the *A*-exciton and significant increase in the intensity of the *B*-exciton are related to the creation of vacancy bands within the band gap as the number of vacancies increases, as discussed above in Fig. 2. In the pristine single layer, the exciton recombination is preferred via VBM-*A*, as the *A*-exciton peak is dominant. In a defect-rich system, however, the in-gap vacancy bands above VBM-*A* could disrupt the recombination routes of the excitons, leading to an increased preference for recombination via VBM-*B*. In other words, the exciton recombination between CBM and VBM-*B* is enhanced, and that between CBM and VBM-*A* is weakened, so PL measurements show the increase of the *B*-exciton signal while the intensity of the A-exciton decreases. As for how such recombination preference occurs after irradiation, we expect that the defect induced in-gap bands play an important role^2^,^43^. But the exact role is complicated by many factors, such as molecular adsorption, temperature, laser power, etc. They could alter the charge carrier dynamics in a complicated way so that it is difficult in the current work to reveal the underlying physical mechanism. A detailed investigation on defect-engineering PL will be the subject of a subsequent study. Finally, we note that the broadening of the *B*-exciton peak may indicate different defect clustering configurations with different binding energies that contribute significantly to the overall intensity of the *B*-exciton peak^2^.

**Figure 5:**
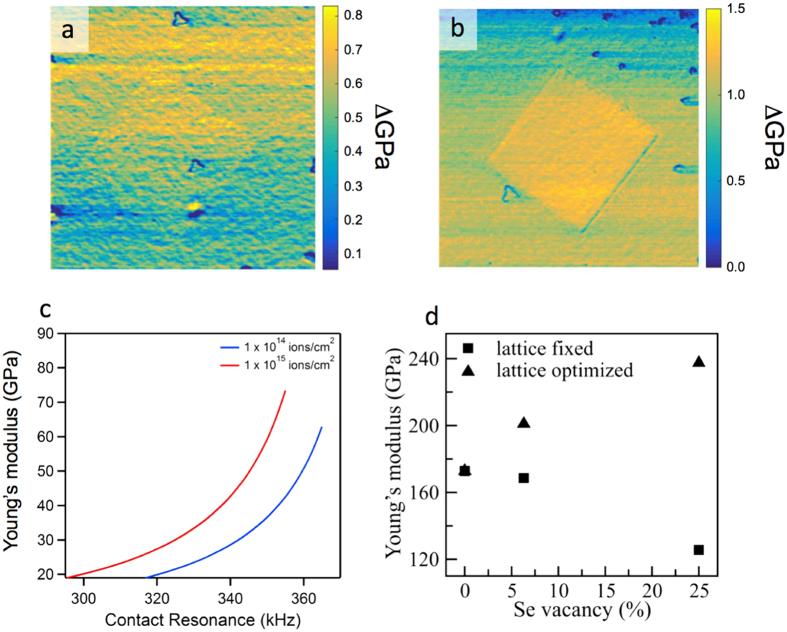
Nanomechanical measurements of supported MoSe_2_ using contact resonance band excitation (BE) scanning probe microscopy. (**a**) Storage modulus maps of supported MoSe_2_ irradiated with 1 × 10^14^ ions/cm^2^ He^+^ beam dose; (**b**) Storage modulus maps of supported MoSe_2_ irradiated with 1 × 10^15^ ions/cm^2^ He^+^ beam dose. (**c**) Young’s modulus of elasticity curves corresponding to He^+^ beam doses of 1 × 10^14^ ions/cm^2^ (blue trace) and 1 × 10^14^ ions/cm^2^ (red trace). (**d**) Calculated Young’s modulus of single layer MoSe_2_ with different Se vacancy concentrations. For each vacancy concentration, two scenarios are considered: lattice constants fixed to the original ones (squares) and lattice constants re-optimized (triangles). After re-optimization, the system size is slightly reduced, suggesting vacancy-induced compression.

Vacancies in single layer MoSe_2_ can influence mechanical performance of the material, as missing atoms change the bond strength and volume of the system^45^. To probe this we performed AFM-based contact resonance measurements to explore nanomechanical properties of the supported MoSe_2_ exposed to the He^+^ beam. In these measurements resonance characteristics of the AFM tip in contact with surface are used to estimate change in the mechanical properties of the sample. In Fig. 5, the storage modulus maps corresponding to regions irradiated with 1.0 × 10^14^ ions/cm^2^ dose (Fig. 5a) and 1.0 × 10^15^ ions/cm^2^ (Fig. 5b) indicate an increase in the elasticity of the MoSe_2_ as the He^+^ dose increases. The Young’s modulus maps in Fig. 5a,b were calculated by converting the frequency shift of the cantilever through the master curves illustrated in Fig. 5c^38^,^46^,^47^. A classical fixed lattice model would suggest a decrease in Young’s modulus, as more defects are introduced, however since vacancies tend to introduce strain – the lattice shrinks. Therefore, after lattice re-optimization, the bond strength in the defect-rich MoSe_2_ is increased compared to the pristine structure (See Supplementary Table 1).

To understand the nanomechanical measurements, we calculated the Young’s modulus *Y*, for pristine and defective single layer MoSe_2_ using the relationship in Equation 1:


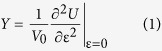


by applying a small uniaxial strain ε, where *V*_0_ is the volume at equilibrium and *U* is the total energy^45^,^48^. *Y* is related to two terms: 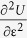 for the system’s bond strength and *V*_0_ for the volume. According to the calculations shown in Table S1, when the lattice is fixed to the pristine one, the increasing vacancy concentration leads to more unsaturated bonds and decreasing bond strength, as reflected by the decreasing term 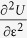 relative to the pristine value. As for the volume, its definition for 2D materials is not entirely clear, particularly for the thickness. A common approach is to take the interlayer spacing in the bulk as the thickness, assuming the system as a uniform slab^49^,^50^. The vacancies in single layer MoSe_2_ can modify the local electronic density distribution and render the uniform slab model inadequate. Therefore, we adopted a new volume definition proposed recently^45^, in which the volume of the single layer is chosen so that the average electronic density inside the volume is equal to that of the parent bulk material. The volume is dependent on the electronic distribution and thus directly includes the geometry of the structure (particularly the vacancy effect)^45^. Our calculations indicate that upon the introduction of Se vacancies, *V*_0_ decreases as well (Table S1), due to missing atoms. With the lattice fixed, 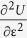 reduces more rapidly than*V*_0_ and the Young’ modulus decreases with the vacancy concentration (squares in Fig. 5d), in line with the general belief. However, the unsaturated bonds destabilize the system, and it tends to shrink slightly for re-stabilization. After the lattice re-optimization, it is found that with larger vacancy concentration, the system is compressed more (Table S1). As a result, 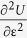 is restored and even increased (Table S1). Combined with the decreased *V*_0_, the Young’ modulus turns out to increase with the vacancy concentration (triangles in Fig. 5d). This suggests that vacancy-induced local strain and reduced volume are potentially responsible for the modulus increase illustrated in Fig. 5a,b.

In summary, we have demonstrated the utility of HIM in tuning electronic and mechanical functionality of the transition-metal dichalcogenide MoSe_2_ through controlled local defect engineering. Our nanomechanical measurements indicate that ion beams can alter the mechanical properties of 2D semiconductors by changing the Young’s modulus of elasticity. BE-KPFM measurements of the surface potential and capacitance gradient suggest that direct-write He^+^ beam lithography can also locally tune the electronic properties of 2D semiconductors with high precision. Finally, PL measurements have demonstrated the capability of the ion beam to locally tune the optical response of MoSe_2_ towards the visible region. As the development of more efficient 2D optoelectronic and semiconducting devices continues, direct-write lithography with He^+^ beams will become an invaluable tool for precision defect-engineering, critical to the development of new functionalities in 2D based devices.

## Methods

### Supported and Suspended MoSe_2_ Synthesis

Two-dimensional MoSe_2_ crystals were synthesized using a method described elsewhere^51^. Briefly, the MoSe_2_ crystals were synthesized by CVD using a tube furnace in a 2-in. quartz tube at a growth temperature of 780 °C. In a typical run, the growth substrates, Si with 250 nm SiO_2_ were cleaned with acetone and isopropanol, placed face-down above an alumina crucible containing ~0.25 g of MoO_3_ powder, which was then inserted into the center of the quartz tube. Another crucible containing ~1.2 g of selenium powder was placed upstream in the tube. After evacuating the tube to ~5 mTorr, flows of 40 sccm (standard cubic centimeter per minute) Ar and 4 sccm H_2_ were introduced into the tube, and the reaction was conducted at 780 °C (with a ramping rate of 30 °C/min) for 10 mins at a total pressure of 20 Torr. At 780 °C, the temperature at the location of the Se powder was ~290 °C. After growth, the furnace was cooled to room temperature.

A uniform and precise amount of stoichiometric nanoparticles were first synthesized and deposited onto a source substrate by pulsed laser deposition (PLD) at room temperature, which was then covered with a TEM grid to form a confined vapor transport growth (VTG) system. By heating the source substrate in an inert background gas, the confined nanoparticles evaporated and grew crystalline 2D flakes on the TEM grid^52^,^53^.

### He^+^ Irradiation of Supported MoSe_2_ Crystal

Supported single layer MoSe_2_ areas corresponding to 1 μm^2^ were irradiated with a 30 kV He^+^ beam in a Zeiss ORION Nanofab helium ion microscope. We used a beam current of ~4.0 pA and dwell time of 50.0 μs per pixel; helium ion doses were 1.0 × 10^14^ ions/cm^2^, 1.0 × 10^15^ ions/cm^2^ and 1.0 × 10^16^ ions/cm^2^. Ion-generated secondary electron (iSE) images of the irradiated crystals were also acquired in order to aid subsequent band excitation scanning probe microscopy and optical measurements.

### Band excitation (BE) Contact Resonance and Kelvin Probe Force Microscopy of MoSe_2_

Band excitation (BE) Kelvin probe force microscopy measurements of the He^+^ beam irradiated MoSe_2_ crystal were performed using an Asylum Research Cypher AFM equipped with BlueDrive. Pt-Cr coated cantilevers were used for the different imaging modes in the AFM. Images were collected at a 2^nd^ cantilever resonance mode of 340 kHz. Band excitation KPFM measurements on the exact same region were collected at the first cantilever resonance mode of 64.25 kHz. The KPFM measurements were performed in interleave mode at a tip-sample height of 50 nm, tip bias of 1 V on a grounded substrate. Post-acquisition image processing was done with WsXM^54^ and MATLAB.

### Photoluminescence (PL) Characterization of Supported MoSe_2_

Photoluminescence (PL) measurements were performed in a Renishaw micro-Raman microscope using a 532 nm excitation laser. A 100X objective lens was used for spectral acquisition with a ~0.6 μm spot size and a 10 s acquisition time. The laser power was approximately 1 mW, which has been chosen to avoid substrate heating and defect diffusion. Data analysis was conducted using WiRE software.

### Scanning Transmission Electron Microscopy (STEM) of Suspended MoSe_2_

Atomic resolution imaging of suspended MoSe_2_ was performed in an aberration-corrected Nion UltraSTEM-100 operating at 60 kV and equipped with a cold field emission gun (CFEG). The convergence semi-angle for the incident electron beam was 31 mrad. Prior to imaging, the sample was baked at 160 °C for 8 hrs under vacuum.

### Local Crystallography and Atom Finding

Local crystallography analysis was performed on the STEM images using methodology described at length elsewhere^30^,^31^,^32^. Briefly, atoms are identified in the STEM image and a local neighborhood of length and angle to the nearest neighbor atoms are constructed. Clustering methods are then employed on the resulting array set utilizing a square Euclidean space metric. Optimal cluster numbers are investigated via a dendrogram as well as oversampling and undersampling the cluster space for the most physically meaningful result.

### Density Functional Theory (DFT) Calculations

Plane-wave DFT calculations were performed using the VASP package^55^ equipped with the projector augmented-wave (PAW) method for electron-ion interactions. The Local Density Approximation (LDA) was adopted for the exchange-correlation interaction with the energy cutoff set at 300 eV^56^,^57^. Single layer MoSe_2_ systems were modeled by a periodic slab geometry and a vacuum separation of at least 18 Å in the out-of-plane direction was used to avoid spurious interactions with replicas. In the 2D slab calculations, all atoms were relaxed until the residual forces were below 0.001 eV/Å. For a primitive hexagonal unit cell of pristine MoSe_2_, its optimized in-plane lattice constant is 3.25 Å and 24 × 24 × 1 k-point samplings were used in the Monkhorst-Pack scheme. To model the electronic properties of different Se vacancy concentrations in single layer MoSe_2_, hexagonal supercells of different sizes were chosen with a single Se atom removed in each supercell. In particular, we have considered a 7 × 7 supercell with a Se vacancy (vacancy concentration 1.0%), a 4 × 4 supercell with a Se vacancy (vacancy concentration 3.1%), and a 2 × 2 supercell with a Se vacancy (vacancy concentration 12.5%). Note that the vacancy concentration is computed as the single Se vacancy divided by the total number of Se atoms in the supercell. For each supercell, the atoms were re-optimized with fine k-point samplings until the residual forces were also below 0.001 eV/Å. Similar procedures were taken to model Mo vacancies.

To study the vacancy effect on the mechanical properties of single layer MoSe_2_, we built rectangular unit cells of different size for uniaxial strain tests. For the pristine MoSe_2_, its rectangular unit cell has a lattice constant 5.63 Å along the armchair direction and a lattice constant of 3.25 Å along the zigzag direction (16 × 24 × 1 k-point samplings were used). From such rectangular unit cell, a 2 × 2 supercell was also constructed. For each system, a single Se atom was removed, leading to different Se vacancy concentrations: 25.0% for the rectangular unit cell and 6.3% for the 2 × 2 supercell. Then for each defective system, the elastic properties were studied in two scenarios: the lattice constants fixed or re-optimized. For the first scenario, only the atoms were relaxed until the residual forces were also below 0.001 eV/Å; for the second scenario, both the atomic coordinates and lattice constants were optimized using the method of fixing the total volume (ISIF = 4 in VASP) to avoid the collapse of the vacuum separation in the *z*-direction^58^. The Young’s modulus *Y* was computed by the application of small uniaxial strains ε (±0.1%, ±0.2%) in the harmonic regime:
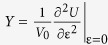
, where *V*_0_ is the volume at equilibrium and *U* is the total energy^45^,^48^. The uniaxial strains were applied in either the armchair direction or zigzag direction, and the Young’s modulus was taken as the average of the values in both directions.

## Additional Information

**How to cite this article**: Iberi, V. *et al*. Nanoforging Single Layer MoSe_2_ Through Defect Engineering with Focused Helium Ion Beams. *Sci. Rep*. **6**, 30481; doi: 10.1038/srep30481 (2016).

## Supplementary Material

Supplementary Information
