# Supplementary material for: Nanoforging Single Layer MoSe2 Through Defect Engineering with Focused Helium Ion Beams
Source: Sci Rep. 2016 Aug 2;6:30481. doi: 10.1038/srep30481 (PMC4969618; doi:10.1038/srep30481)
Supplement: Supplementary Information [file srep30481-s1.doc]

**SUPPLEMENTAL MATERIALS**

**Nanoforging Single Layer MoSe_2_ Through Defect Engineering with Focused Helium Ion Beams**

*Vighter Iberi^1,2*^, Liangbo Liang^1^, Anton Ievlev^1,3^, Michael G. Stanford^1,2^, Ming-Wei Lin^1^, Xufan Li^1^, Masoud Mahjouri-Samani^1^, Stephen Jesse^1,3^, Bobby G. Sumpter^1,3^, Sergei V. Kalinin^1,3^, David C. Joy^1,2^, Kai Xiao^1^, and Alex Belianinov^1,3^, Olga S. Ovchinnikova^1,3*^*

1. Center for Nanophase Materials Sciences, Oak Ridge National Laboratory, Oak Ridge, TN 37831, USA.

2. Department of Materials Science and Engineering, University of Tennessee, Knoxville, TN 37996, USA.

3. The Institute for Functional Imaging of Materials and the Center for Nanophase Materials Sciences, Oak Ridge National Laboratory, Oak Ridge, TN 37931, USA.

| **MoSe_2_** | | 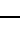 **(eV)** | 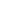 **(Å^3^)** | 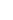**(GPa)** |
| --- | --- | --- | --- | --- |
| **6.3% vacancy** | **lattice fixed** | **471.3** | **447.9** | **168.6** |
|  | **lattice optimized**  **(reduced by ~1.3%)** | **562.5** | **448.4** | **201.0** |
| **25.0% vacancy** | **lattice fixed** | **79.1** | **100.9** | **125.6** |
|  | **lattice optimized**  **(reduced by ~4.6%)** | **150.0** | **101.2** | **237.5** |
| **Pristine counterpart** | | **124.8** | **115.7** | **172.8** |

Table S1. Modeling parameters of MoSe_2_ Young’s modulus with different concentration of Se vacancies.


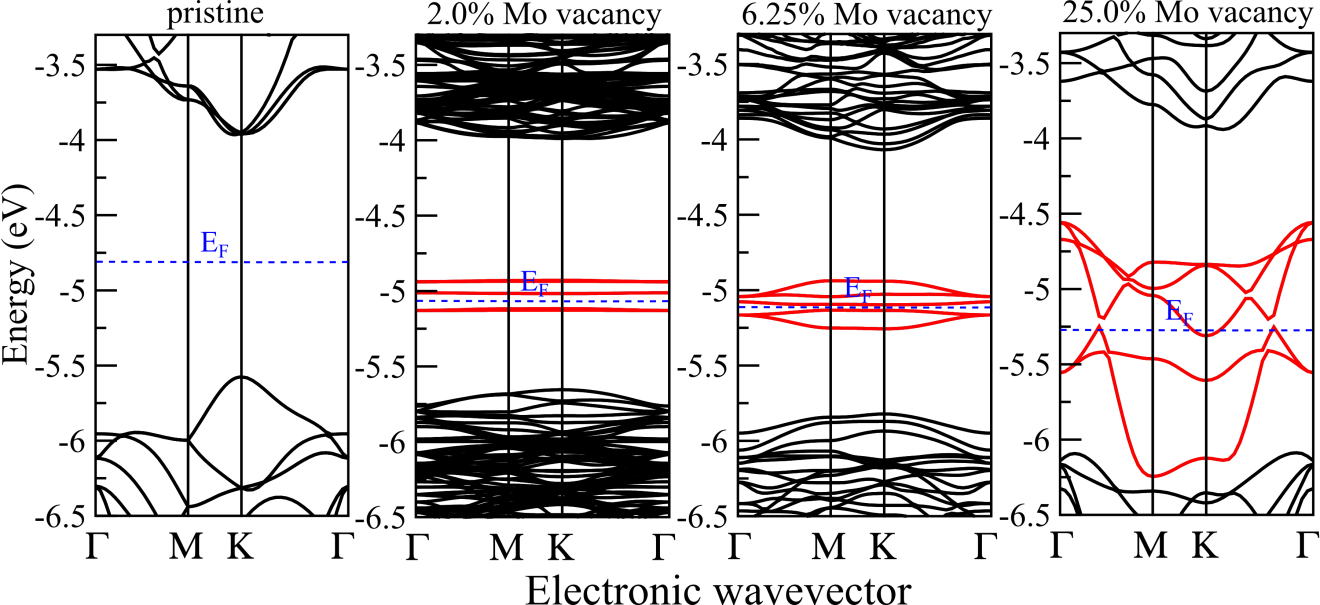


Figure S1: Calculated electronic band structures of single layer MoSe_2_ with different Mo vacancy concentrations. All band energies are aligned to the vacuum potential for direct comparison. The Mo vacancy induced in-gap bands are highlighted in red color. The Fermi level is set at the middle of the band gap for each system, as shown by the blue dash line.

Compared to Se vacancies illustrated in Figure 2 in the main text, a distinctly different feature of Mo defects is that the vacancy induced in-gap bands are much closer to the Fermi level, rendering the system’s band gap much smaller, as shown in Figure S1. The band gap of the Mo defective system is decreasing with growing Mo vacancy concentration and it eventually becomes metallic. But our PL measurements suggest that the sample retains the semiconducting properties (though weakened), this also indicate that Mo defects are not dominant.

Despite the band gap differences between Mo-defective and Se-defective systems, for both cases, our calculations suggest that the Fermi level of the system is continuously downshifted with the increasing vacancy concentration; due to the vacancy induced in-gap bands (see blue dashed lines in Figure 2 and Figure S1). Consequently, the work function of the defective system is higher than that of the pristine one, and furthermore the work function slightly increases with the growing vacancy concentration. This calculated trend is in line with our KPFM measurements in Figure 3 that the work function increases slightly with the increasing dose (i.e., larger vacancy concentration).
